# Innovating New Rural Cooperative Medical Scheme (NCMS) for Better Patient Satisfaction in Rural China

**DOI:** 10.3390/ijerph15092007

**Published:** 2018-09-14

**Authors:** Dongxiao Gu, Xuejie Yang, Xingguo Li, Changyong Liang, Jinhong Zhong, Nanping Feng

**Affiliations:** 1 The School of Management, Hefei University of Technology, Hefei 230009, China; gudongxiao@hfut.edu.cn (D.G.); xuejie_Y@126.com (X.Y.); lixingguo@hfut.edu.cn (X.L.); jinhong.zhong@hfut.edu.cn (J.Z.); fengnp@hfut.edu.cn (N.F.); 2 The School of Informatics, Computing and Engineering, Bloomington, IN 47405-3907, USA

**Keywords:** New Rural Cooperative Medical Scheme systems, patient satisfaction, perceived usefulness, health service quality, rural places in China

## Abstract

With the broadening application of the New Rural Cooperative Medical Scheme (NCMS), its role in patient satisfaction in rural China has shifted to be the focus of academic research. Based on a technology acceptance model, this study will investigate the factors and mechanisms influencing patient satisfaction on NCMSS in rural places in China. In this study, based on a technology acceptance model, we developed a model that is associated with the influencing factors, patients’ continued participation and patient satisfaction, and conducted an empirical analysis based on data collected from rural areas of China’s Anhui Province. A NCMS′s reputed reliability, value, and convenience played a key role in positively influencing patient satisfaction. However, long-term patient participation was not significantly influenced by other social factors. In order to increase patient satisfaction, NCMS policy and implementation procedure needs further government modification and innovation with the goal of improving the reimbursement ratio, reducing the financial burden, and improving patient convenience.

## 1. Introduction

For rural residents in China, subsidizing healthcare costs through insurance schemes can avoid high medical expenditures and overcome financial barriers to healthcare service [1,2]. Fifteen years ago, China introduced a new community-based rural health insurance system, named the New Rural Cooperative Medical Scheme (NCMS). NCMS is a voluntary insurance scheme designed mainly for rural residents. Financed with the cooperation of individual, local, and central governments, it has a risk pooling unit in one rural county [3]. By the end of 2014, 98.9 % rural residents (approximately 736 million) in China had joined the NCMS [4]. It plays a very important role in Chinese rural residents’ health care and it has raised concerns for health service quality and drug-use safety in rural China [4]. Subsidizing healthcare costs through NCMS is not only crucial to overcoming financial barriers to healthcare and avoiding high medical expenditures, but it has also proved to be helpful in decreasing financial risk for rural residents. These have been achieved primarily by decreasing out-of-pocket payments [2,5,6]. Although NCMS undoubtedly benefits hundreds of millions of rural Chinese patients, NCMS implementation has still encountered many problems. 

One key concern is whether or not more insurance coverage would lead to improved customer service and customer health benefit. While this question cannot be easily solved, it has been clear that patient satisfaction—especially for NCMS rural rural residents—indeed has great room for improvement. Hence, whether NCMS should be redesigned with incentives for the improvement of health service quality and patient satisfaction has become a key issue in the current debate over China’s recent health care reform. In recent years, China has conducted a new round of health care reform, and it has extended public health insurance coverage in order to improve the health of rural residents [7]. Implementation of modified NCMS has to some extent improved the level medical insurance in rural places, by providing patients with convenient health care at a lower cost. NCMS has substantially improved health care access and utilization among the participants [8], and it has proved to be effective in reducing medical impoverishment in China′s low-income regions [9]. With the growth of NCMS financing and coverage, the relationship between NCMS and its patients needs to be reoriented, and it is important to understand the assessment and satisfaction of patients on the use of NCMS. 

NCMS patient satisfaction refers to the difference between patient expectations and actual patient experience. Recently, patient satisfaction with NCMS has become an important index for measuring the quality of health insurance services in rural Chinese, and it plays an important role in the improvement of rural primary healthcare insurance service [10]. In order to discover and solve problems within the system, it is important to learn about actual patient experiences [11]. With the increasing presence of NCMS in rural China, urban and rural social development research must change to include consideration of patient satisfaction. The core challenge is to explore the factors that affect patient satisfaction on NCMS, as well as key determinants and their influential mechanism on patient satisfaction [12]. 

Using technology acceptance model (TAM) in combination with perceived reliability (PR) and other social factors, we investigated the influences found on patient satisfaction. In the next section, we will review previous literature. In Section 3, we will develop our hypothesis and construct our research model. We introduce the scales used for the construction of our model and the data collection procedure in Section 4 and report the results of data analysis in Section 5. In Section 6, the study is concluded and some discussion is offered. 

## 2. Literature Review

The concept of patient satisfaction is derived from customer satisfaction [13]. The generally acceptable definition of customer satisfaction is consumers’ affective assessment on service or products during or after the purchasing process. People evaluate their satisfaction by comparing the actual product or service with their previous expectations for the product or service. If the actual experience with the product is better than the expectation, then the customer is satisfied. If not, the customer is dissatisfied. While patient satisfaction is an important outcome measure for health services, both the nature of patient satisfaction and the meaning of expressions of ‘satisfaction’ are hard to define. Currently, there is still no accurate definition for patient satisfaction. However, most of the research contains both explicit and implicit aspects. Explicit satisfaction is the effect of medical service, while implicit satisfaction is the experience of receiving both insurance and medical service [14]. Schoenfelder et al. found that interpersonal relationships between doctors and nurses, action organization, admission and discharge, and perceived stay time affect patient satisfaction [15]. Wang et al. not only studied the impact of factors, such as demographic information on patient satisfaction, but also found that the sustainability of the new rural cooperative medical plan has an impact on the overall satisfaction of the plan [16]. Tasso et al. measure patient satisfaction through patient interviews and participant observations [17]. In the study of patient satisfaction, scholars mostly use interviews and field research methods. Personal factors, such as gender, age, and physical condition are often used to verify the impact on patient satisfaction. There is currently no systematic study of factors affecting patient satisfaction. This paper establishes a model to systematically study the impact of patients on the continued use and satisfaction with CMS.

## 3. Model and Hypotheses Development

We found several theories supporting our current study. The main one is TAM, which is founded on the theory of reasoned action (TRA) [18] and the theory of planned behaviour (TPB) [19]. TAM was first used by Davis in order to explain the decision factors that influence acceptance of new information technologies [20]. To describe conscious behaviour, Fishbein and Ajzen proposed the theory of reasoned action (TRA) [21], which is based on social psychology. TRA measures the intention of spontaneous behaviour from the perspective of social psychology, indicating that personal attitudes towards specific behaviour depend on cognitive differences. These differences are based on both social and personal factors [22].

TPB was developed from TRA. Ajzen [18] believes TRA is weak to explain and predict the behaviour, since it is too limited in its sources. He therefore proposes TPB as an expansion of the explanation of human behaviour. In TPB, past actions will affect intentions. In reality, the resulting behaviour sometimes requires factors, such as personal ability, knowledge, and help from others, as well as resources that do not have a close relationship with personal motivation. Before the formation of the will, these factors are called individual perceived behavioural control and represent the expected ease or difficulty of producing an action. 

Davis [23] uses TRA to investigate the relationship between cognitive emotional factors and technology use and proposes TAM. This model includes two main factors: perceived usefulness and perceived ease of use. Davis believes that the use of a particular system depends on the intended behaviour of an individual user, and that intention is determined by the attitude and perceived ease of use. Attitude is decided by perceived usefulness and perceived ease of use. Both perceived usefulness and perceived ease of use are determined by external variables [24]. TAM has been introduced in the research of various health technologies, as well as other health products and health service [25,26,27].

**Figure 1:**
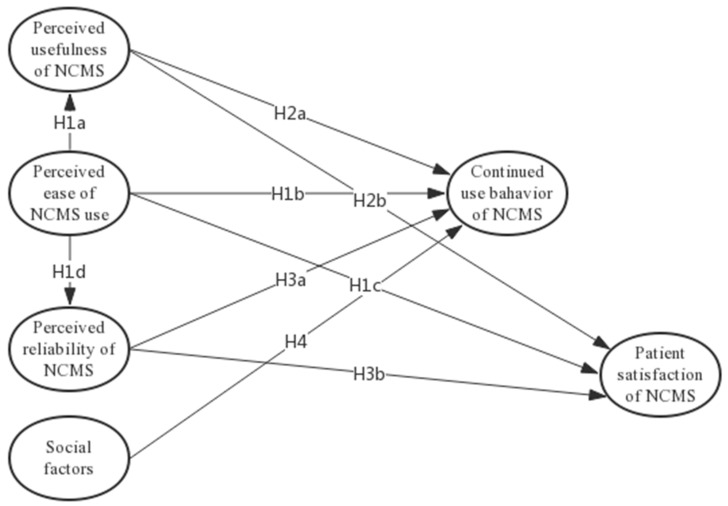
Research Model.

Based on the above theory, this paper establishes a model to study the impact of perceived usefulness (PU), perceived ease of use (PE), perceived reliability (PR), and social factors on continued use behavior (CUB) of NCMS and patient satisfaction (PS) with NCMS. The research model for this study is shown in Figure 1.

### 3.1. PU, PE and CUB, PS

NCMS has covered most of rural Chinese residents, and it has greatly improved the level of rural public health and social security in China [28]. Millions of rural residents in China joined NCMS and now use it for the reimbursement of their medical expenses or drug costs generated in hospitals, community health service agencies or pharmacy stores [29]. The ultimate goal of rural NCMS patients is to obtain better health service and better medical insurance security. Therefore, they will continue to participate NCMS and feel satisfied only when they consider it useful and convenient [30,31]. The easier it is to use the NCMS, the stronger the perceived usefulness will be, and the greater their willingness will be to become NCMS’ satisfied participants and long-term users. Hence, the hypothesis:

**H1a:**  *Perceived ease of use of NCMS has a significant positive impact on the perceived usefulness of NCMS.*

**H1b:**  *Perceived ease of use of NCMS has a significant positive impact on the continued use behaviour of NCMS.*

**H1c:**  *Perceived ease of use of NCMS has a significant positive impact on the patient satisfaction of NCMS.*

**H2a:**  *Perceived usefulness of NCMS has a significant positive impact on the continued use behavior of NCMS.*

**H2b:**  *Perceived usefulness of NCMS has a significant positive impact on the patient satisfaction of NCMS.*

### 3.2. PR and CUB, PS

In this study, perceived reliability (PR) contains two aspects, namely, perceived risk and perceived trust. The perceived risk proposed by Bauer [32] extends from psychology. He believes that it is unlikely that the result of a predicted purchase is certain, and that some of the results may make the customer feel unpleasant. Therefore, a consumer purchase decision is impacted by the uncertainty of the results. This is the original concept of risk. Malhotra [33] defined perceived risk as the expectation of a high potential for loss, and found that trusting/risk are expected to exert significant effects on behaviour. Heijden [34] defined perceived risk as subjective perceptions of negative consequences, as well as the probability that the negative consequences may occur after the purchase of products. Research shows that attitude and trust, defined as the antecedent of perceived risk, and technology, defined as the antecedent of perceived ease of use, directly influence online purchasing. Green & Pearson [35] found that perceived risk reduction can increase continued use. Gefen et al. [36] suggests that the perception of trust had a significant impact on user behaviour.

For rural residents in China, enrolment in NCMS is optional and fully autonomous. Although NCMS is supported by the central and local governments, participants must generally pay some of the medical insurance premiums. The relative insufficiency of insurance funds results in relatively low government repayment accounts. In some situations, the majority of health care expenditures must be paid by the patients who registered in NCMS. This could affect both the patients’ perceived risk and their ability to trust NCMS. The implementation status of NCMS has also affected the satisfaction of rural residents. Meanwhile, due to information asymmetry and relatively complicated reimbursement procedures, some rural residents do not know which medical expenses are eligible for reimbursement. Hence, although a rural household may participate in NCMS, they may still refrain from using required health services. This is especially common when the situation involves costly diseases, since the patients worry that high medical expenses could drive the household into poverty. For some low-income households, high medical cost put them at a particularly high risk of falling into poverty. Hence, we hypothesize:

**H1d:**  *Perceived ease of use has a significant positive impact on the perceived reliability of NCMS.*

**H3a:**  *Perceived reliability has a significant positive impact on the continued use behaviour of NCMS.*

**H3b:**  *Perceived reliability has a significant positive impact on the patient satisfaction of NCMS.*

### 3.3. SF and CUB, PS

Social factors are a kind of social influence that will lead to individual behavior, such as technology adoption, service use, and decision making. Understanding the influence of an individual’s friends, relatives, acquaintances, neighbours, and classmates on behavior has been of interest to scholars in a wide variety of fields [37]. Venkatesh et al. [38] found that, in using a particular system or service, individuals will be affected by the views of the people around them. Individual behaviour will be affected by others in their social networks. Social factors, such as the attitudes of their people around the rural residents, will affect their continued use of NCMS. For rural residents in China, the surrounding social factors is also an important factor affecting whether or not they continue to participate in NCMS. The continued use of NCMS by rural residents will probably be affected by the social environment in which they are staying in. In the belief elicitation phase, the participants identified four specific groups of people who are likely to influence the usage of NMCS behavior. These were relatives, acquaintances, friends, and neighbours. Social environmental factors could influence individual intention or behavior by changing their psychology and thinking [39]. Therefore, we hypothesize:

**H4:**  *Social Factors have a significantly positive impact on the continued use behaviour of NCMS.*

## 4. Methodology

### 4.1. Measures

Measures for all of the variables were adapted from previous studies. Six variables were measured in this study: Perceived Usefulness (PU), Perceived Ease of Use (PE), Continued Use Bahavior (CUB), Perceived Reliability (PR), Social Factors (SF), and Patient Satisfaction (PS). The items were measured with a seven-point Likert scale, ranging from 1 (strongly disagree) to 7 (strongly agree). The measures for the variables are shown in Table 1. Perceived usefulness of NCMS (PU) and perceived ease of NCMS use (PE) are, respectively, measured by a four-item scale adapted from Yoon [40]. The construct of social factors (SF) are measured by a four-item scale adapted from Limayem [41]. To measure perceived reliability (PR), we used a five-item scale that was adapted from Gefen et al. [42,43,44,45]. Continued use behavior of NCMS (CUB) is measured by a three-item scale adapted from Sun [46]. Patient satisfaction was measured by a five-item scale from Liang [47]. Based on the above scales, we developed a survey instrument. After compiling the English version of the instrument, the items were first translated into Chinese by a young bilingual researcher, and then verified, refined, and back-translated for translation accuracy by a professor in research area of rural medical insurance and public health.

### 4.2. Data Collection

Our research survey was generally met with the full support of local residents. The data was collected in randomly selected villages in rural parts of Anhui Province. The participants are from six countries in Anhui Province. We collected the data using a multistage iterative process. The first country is located in Huaihe River Basin and affiliated with Huinan City, Anhui Province. Five villages from this country were randomly selected for survey. Three of them are close to Hefei, the capital of Anhui Province. These three villages each were home to approximately 290, 330, and 420 residents, respectively. The fourth is approximately 70 miles away from Hefei, and it contains 390 people. The last one has approximately 320 residents. The second country is affiliated with of Hefei. Amongst the four villages, three are located in the north of this country with approximately 320, 370, and 440 residents, respectively. The last is located in the south, is close to Hefei, and contains approximately 450 residents. The third country is located in the Huaihe River Basin and Central Anhui Province, affiliated to Chuzhou city. Amongst the three villages, two are approximately 45 miles far away from the capital of country and have approximately 350 and 310 residents. The third has 270 residents. The fourth country is affiliated with Huaibei City and is located in North Anhui Province. The three randomly selected villages are about 35 miles away from the capital of the country and have approximately 300, 280, and 290 residents. The fifth country is located in the Dabie Mountain Area of West Anhui Province. Amongst the two randomly selected villages, one is less than ten miles away from the capital of the country and contains over 230 residents, and the other is approximately 70 miles away from the capital of country with 260 residents. The former village has a relatively developed economy, while the latter is relatively poor. The sixth country is located in the Yangtze River Basin of South Anhui Province. Amongst two randomly selected villages, one is approximately fifty miles away from the capital of the country and it contains about 210 residents, while the other is approximately 100 miles away from the capital of country with approximately 170 residents. Both of these villages are located in the 1000 m mountain area (Dabie Mountains) and they are relatively undeveloped. Ten percent of the villagers were randomly selected as participants to fill in the questionnaires.

First, we adapted the original measures from literature to the healthcare context. Next, we translated survey into Chinese using a professional translation staff. Second, we conducted a pilot study with 75 respondents, with the goal of improving ambiguous expressions, awkward wordings, and distortions of the original meanings. Based on the data and respondent suggestions during the pilot study, we modified the questionnaire. The modified questionnaire was then used to collect data from participants in rural areas of Anhui Province, China. We gave respondents a small gift for completing and returning the questionnaire. The questionnaires were randomly distributed to participants. Rural residents who could not read or write were not selected to participate in the study. We took a few precautions in order to avoid selection bias as much as possible. Our choice of countries in Anhui Province was made randomly by a computer program. From those countries, 19 villages were randomly selected. Finally, the participants were randomly selected. A total of 600 questionnaires were distributed and 590 questionnaires were returned, yielding a response rate of 98.33%. After removing invalid questionnaires (incomplete, repeated answers, obvious contradictions, etc.), we obtained 483 valid questionnaires, yielding an effective response rate of 81.86%. Table 2 presents the demographic characteristics of the respondents in this study.

## 5. Results

### 5.1. Measurement Model

The means and loadings of each measured item and the descriptive statistics of each item are given in Appendix A. The loadings of all the items were above the threshold of 0.75, indicating that the observed variables had high convergent validity. Additionally, the loadings showed a high correlation between observed and structural variables [48].

The acceptability of the measurement model was assessed by the reliability of individual items, internal consistency between items, and the model’s convergent and discriminant validity. *SmartPLS 3.0* (SmartPLS GmbH, Hamburg, Germany) was employed to assess the measurement model. Table 3 shows the composite reliability, average variance extracted (AVE), and the square root of AVE, as well as the correlations between constructs. Scale reliability is an important measure of scale adequacy. When scale reliability is high, variables measuring a single factor share a high degree of common variance [49]. The Cronbach’s alphas of the seven constructs were all above the recommended criterion of 0.70 [50], ranging from 0.8211 (perceived usefulness) to 0.8847 (continued use behavior), showing that the measures were internally consistent. The composite reliability values of all the constructs were above 0.8, exceeding the cut-off value of 0.70 [51], which indicated adequate internal consistency [52]. The AVE for each construct was higher than 0.50, suggesting that the observed items explained more variance than the error terms [53]. Additionally, the square root of the AVE for each construct was higher than the correlations between the construct and all other constructs, suggesting excellent discriminant validity. The results show that all scales of the measurement model demonstrate adequate internal consistency for further analysis of the construct model.

### 5.2. Structural Model

**Figure 2:**
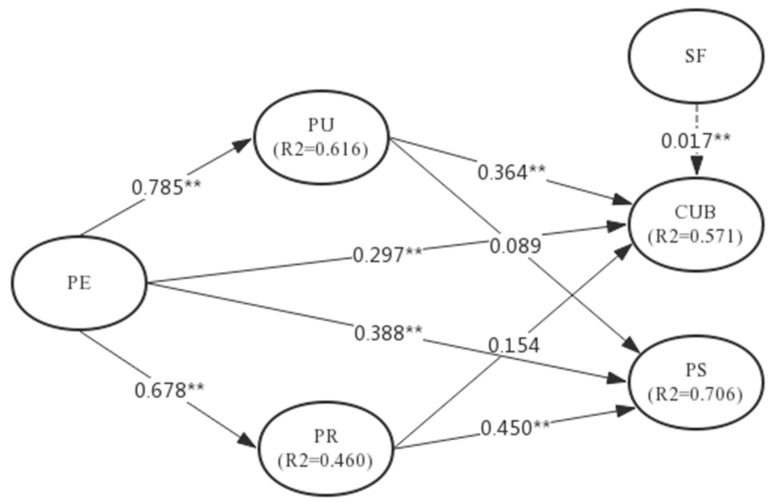
Model Results. Path coefficients with t-values in parentheses, ** *p* < 0.01.

Structural equation modelling was applied to analyse data while using the partial least squares method using *SmartPLS 3.0*. The parameter estimated in a structural model exhibited the direct effects of one construct on the other. Therefore, a significant coefficient at a certain level of α reveals a significant relationship between latent constructs (Figure 2, Table 4).

In this study, we use Bootstrapping (*n* = 5000) to perform the significance tests of hypotheses. The results are shown in Table 4.

As shown in Figure 2, the comprehensive effect R^2^ of patient satisfaction was 0.706, thus explaining 70.6% of the variance in patient satisfaction. The comprehensive effect R^2^ of the exogenous latent variables on NCMS continued use reached 0.571, thus explaining 57.1% of the variance in continued use. The comprehensive effect R^2^ of perceived ease of use on perceived usefulness reached 0.616, thus explaining 61.6% of the variance in perceived usefulness. The comprehensive effect R^2^ of perceived ease of use on perceived reliability reached 0.46, thus explaining 46% of the variance in perceived reliability. Therefore, the study concluded that the variables were fully explained.

According to the results in Table 4, hypotheses H1a, H1b, H1c, H1d, H2a, H2b, H3a and H3b were all supported. However, a significant influence of social factors on the constant participation and use of NCMS was not observed. Therefore, hypothesis H4 was not supported. In the next section, we will present a detailed discussion of these findings.

## 6. Discussions

Given the widespread use of NCMS, this study investigated the factors influencing rural residents’ continued participation and use of NCMS, as well as how these factors affect patient satisfaction. Our empirical research results have shown that both perceived usefulness and perceived ease of use have a positive influence on the continued use of NCMS and patient satisfaction. These results are consistent with research that was conducted by Pan et al. [30]. Our research results are also consistent with Yan et al. [31] By studying the relationship between the convenience of NCMS and patient satisfaction, we found that both perceived usefulness and perceived ease of use have significant effects on patient satisfaction. This is an important theoretical contribution and it make that how PE, PR, and PU affect CUB and PS clear PR is a completely variable with new meaning. Meanwhile, our study is also the first one that examine the relationships between PE and PR, between PR and CUB, between PR and PS in public health context. Our study also provides novel perspective to examining and evaluate the effect of NCMS from patients’ angle. To improve continued use, the content systems of NCMS and implementation procedure should be redesigned and simplified. This will ultimately achieve the goal of improving patient satisfaction. The government should also realize that only when the NCMS is more convenient and effective will the satisfaction of patients in rural China see real gains.

Based on the TAM, this study also introduces two factors, namely perceived reliability and social factors, both of which better reflect the patient situation. The results show that perceived reliability impacts the continued use of NCMS and thus could improve patient satisfaction. Our findings are consistent with the conclusion by Tung et al. [54], which was conducted regarding adoption of an electronic logistics information system in the medical environment. Therefore, the government should enhance information publicity and provide better information services. This could include guidance on how to use NCMS and information as to which costs are eligible for reimbursement. Both would reduce perceived risk and uncertainty concerning NCMS. This would improve the perceived reliability of NCMS, and it would encourage them to participate in NCMS. This would be very helpful in promoting rural residents’ health, and would assist in creating sustainable development in these rural areas. In this study, the influence of social factors was found to be insignificant. This inconsistency may be due to the difference of environment: NCMS is used in health, not in business. Undoubtedly, NCMS is very important and useful in public health, and particularly in rural China. 

According to our survey on patient satisfaction (we have a special item to measure if a participant thinks NCMS is beneficial to the quality of care, see Table 1), a majority of NCMS users agreed that NCMS is helpful for them in obtaining a high quality of care. According to further interviewing with the participants, we acquired the following reasons behind their opinion: (1) NCMS can cover part of their healthcare expenditures, which gives patients more confidence in going to big hospitals; (2) Some NCMS users think that doctors will provide better care to patients in NCMS than those patients who are poor and pay independently. From this perspective, NCMS is an important security mechanism both to patients and to hospitals, and it is generally beneficial to the improvement of healthcare quality in China.

Meanwhile, given the increasing demand for higher quality healthcare, NCMS has great cause—and potential—to improve. The Chinese central and local governments are now promoting the Healthy China Initiative, in which the improvement of effectiveness of NCMS and urban and rural residents’ satisfaction is a key target. Current out-of-pocket medical payments remain a burden for most rural households. In some rural areas, in fact, NCMS failed to reduce the financial burden of outpatient medical expenditure of poor communities. This was especially true of those patients with chronic diseases. Generally, financial protection against high healthcare expenditures was rarely present in the care for poor rural residents. Although the nominal reimbursement ratio of inpatient services within the benefit packages had been set as 70% in township hospitals in the modified NCMS policies, the effective reimbursement ratio (ERR) is still not high in some counties, and out-of-pocket medical payment is still high for many rural residents. 

To improve the participation and satisfaction of patients from rural areas, stable and sustainable financing and reimbursement ratio adjustment mechanisms for medical insurance should be provided and improved. The policy of NCMS should be adjusted, and the reimbursement ratio should be further improved. Medical expenditures that are related to serious illness, such as cancers and outpatient services for chronic diseases, should be covered better by the NCMS. The insurance system covering serious illnesses, both for urban and rural residents, should be implemented in all the rural villages as soon as possible. Even emergency health care would be improved for these patients. NCMS should also adopt a coverage policy for chronic conditions as soon as possible. 

Meanwhile, to improve patients’ perceived ease of use of NCMS, the government should provide better information service and accelerate basic medical insurance. This would improve medical treatment and it would provide direct medical expense reimbursement for in-patients. Moreover, it is also suggested that the government departments adjust medical insurance management and payment methods, achieve a sustainable balance of medical insurance funds, and provide stable and reliable insurance security to rural Chinese patients.

This study has some limitations that should be addressed in the future. First, to facilitate the investigation, we use willingness to continue to use NCMS instead of the actual behaviour. In fact, the continued use behaviour deserves more attention. Second, this study only analyzed individual differences, and it did not explore the impact mechanisms of NCMS input on patient satisfaction in different populations (age, gender, education). Finally, in this study, the effects of social factors on the continued use of NCMS have not been verified. Further study should focus on this relationship and discover the reasons.

## 7. Conclusions

In this study, we developed a model examining the influence of perceived ease of use, perceived usefulness, perceived reliability and social factors on the output (CUB and PS) of NCMS based on TAM, TPB and the theory of post-adoption [55].The results of data analysis showed PE had significantly positive effect on both CUB and PS. PU had significantly positive effect on CUB, but had no significantly positive effect on PS. PR had significantly positive effect on PS, but had no significantly positive effect on CUB.PE also had significantly positive effect on PU and PR. SF had no significantly positive effect on CUB.

At present, China is vigorously promoting “*Building a newly socialist countryside*” plan and rural health care reform. How to improve rural medical insurance system is one of important issues of this reform. The results of this study can provide a reference for decision-making for the improvement and optimization of new era rural health care reform in China.

## Figures and Tables

**Table 1:** Measures of Variables.

Measures of Variables		Reference
Perceived Usefulness	As a rural household, NCMS is useful to protect me from the financial risk posed by health care costs. NCMS makes me not worry much about a big medical bill during seeking medical service and can save me much money. NCMS enables me to get medical care timelier. NCMS can save me money in medical care.	[40]
Perceived Ease of Use	It is easy to participate in NCMS. Learning to using NCMS is easy. My interaction with NCMS is clear and understandable. The procedure of NCMS is very simple.	
Social Factors	My relatives think it is important to participate in NCMS. My friends around me think it is important to participate in NCMS. My family members think it is important to participate in NCMS. My neighbours think it is important to participate in NCMS.	[41]
Perceived Reliability	I believe that NCMS is dependable and trustworthy. I believe that NCMS provides good service The level of my trust for NCMS is very high. I trust the NCMS administrators to keep my personal information safe and will not share or sold it to companies for business purpose. I trust the NCMS administrators will not misuse my personal information.	[42,43,44,45]
Continued use behaviour	I was participated in NCMS in the past 12 months. I recommended NCMS to my friends or other people to whom I am familiar. When I seek medical care, I used NCMS. I continued to use NCMS for medical risk protection.	[46]
Patient satisfaction	I am satisfied for that NCMS increase my use of health care services. I feel the NCMS is more consistent with my expectation With NCMS, I feel I am more satisfied with the healthcare in the hospital. NCMS is generally beneficial for the improvement of the quality of care, and I like it. With NCMS, I feel the out-of-pocket spending on medical care are more reasonable.	[47]

**Table 2:** Sample Demographics.

Item	Category	N	%
Sex	Male	229	47.41
	Female	254	52.59
Age	18–28 years old	104	21.53
	28–48 years old	164	33.95
	48–60 years old	97	20.09
	>60 years old	118	24.43
Education background	Primary school	68	14.08
	Middle school	277	57.35
	High school	124	25.67
	College or other	14	2.90

**Table 3:** Measurement Model Results.

Construct	C.A.	C.R.	AVE	CUB	PE	PR	PS	PU	SF
**CUB**	0.8847	0.9293	0.8146	**0.9025**					
**PE**	0.8279	0.8854	0.6591	0.7124	**0.8118**				
**PR**	0.8703	0.9062	0.6596	0.6156	0.6782	**0.8121**			
**PS**	0.8701	0.9059	0.6585	0.6254	0.6984	0.7736	**0.8115**		
**PU**	0.8211	0.8818	0.6513	0.6980	0.7846	0.6787	0.7631	**0.8070**	
**SF**	0.8214	0.8822	0.6523	0.5673	0.6479	0.7873	0.7371	0.6519	**0.8076**

C.A. = Cronbach’s alphas. C.R. = Composite Reliability. AVE = Average Variance Extracted. CUB = continued use behaviour. PE = perceived ease of use. PR = perceived reliability. PS = patient satisfaction. PU = Perceived Usefulness. SF = Social Factors. The bold numbers on the diagonal are the square roots of the variance shared between the constructs and their measures. Off-diagonal elements are correlations among constructs. For discriminate validity, diagonal elements should be larger than off-diagonal elements.

**Table 4:** Structural parameter estimates.

Hypothesized Path	Standardized Path Coefficients	t-Value	*p*-Value	Results
H1a: PE → PU	0.785	30.929	*p* < 0.01	Supported
H1b: PE → CUB	0.364	6.081	*p* < 0.01	Supported
H1c: PE → PS	0.089	1.790	*p* < 0.01	Supported
H1d: PE → PR	0.678	21.440	*p* < 0.01	Supported
H2a: PU → CUB	0.297	5.508	*p* < 0.01	Supported
H2b: PU → PS	0.388	7.436	*p* < 0.01	Supported
H3a: PR → CUB	0.154	2.400	*p* < 0.05	Supported
H3b: PR → PS	0.450	9.517	*p* < 0.01	Supported
H4: SF → CUB	0.017	0.287	Not significant	Unsupported

## Appendix A

**Table A1:** Descriptive Statistics of the Measures.

Construct	Item Statistics			
	Construct Items	Mean	Std. Deviation	Loading ^1^
Perceived Usefulness of NCMS(PU)	PU01	6.11	1.102	0.8301
	PU02	6.03	1.128	0.8182
	PU03	5.92	1.169	0.8220
	PU04	5.85	1.195	0.7553
Perceived Ease of NCMS Use(PE)	PE01	5.73	1.285	0.8143
	PE02	5.76	1.221	0.7841
	PE03	5.88	1.194	0.8425
	PE04	5.66	1.287	0.8053
Perceived Reliability of NCMS(PR)	PR01	5.62	1.366	0.7938
	PR02	5.39	1.461	0.7496
	PR03	5.47	1.301	0.8057
	PR04	5.70	1.194	0.8504
	PR05	5.91	1.179	0.8565
Social Factors(SF)	SF01	5.55	1.377	0.7575
	SF02	5.64	1.422	0.7845
	SF03	5.53	1.449	0.8496
	SF04	5.64	1.323	0.8355
Continued use of NCMS(CUB)	BI01	5.69	1.224	0.9084
	BI02	5.60	1.343	0.8421
	BI03	5.70	1.273	0.9539
Patient Satisfaction with NCMS(PS)	PS01	5.82	1.199	0.7538
	PS02	6.04	1.101	0.8069
	PS03	5.89	1.186	0.8349
	PS04	6.08	1.076	0.8464
	PS05	5.72	1.225	0.8127

^1^ The loading is reported by *SmartPLS 3.0*.

## References

[1] Liang Y., Lu P. (2014). Medical insurance policy organized by Chinese government and the health inequity of the elderly: Longitudinal comparison based on effect of New Cooperative Medical Scheme on health of rural elderly in 22 provinces and cities. Int. J. Equity Health.

[2] Dong H., Duan S., Bogg L., Wu Y., You H., Chen J., Ye X., Seccombe K., Yu H. (2016). The impact of expanded health system reform on governmental contributions and individual copayments in the new Chinese rural cooperative medical system. Int. J. Health Plan. Manag..

[3] Li C., Hou Y., Sun M., Lu J., Wang Y., Li X., Chang F., Hao M. (2015). An evaluation of China’s new rural cooperative medical system: Achievements and inadequacies from policy goals. BMC Public Health.

[4] Filipski M.J., Zhang Y., Chen K.Z. (2015). Making health insurance pro-poor: Evidence from a household panel in rural China. BMC Health Serv. Res..

[5] Ying Y., Pan W., Zhang Z. (2015). Increasing insurance protection efforts and improving the fairness of health care services: A-nalysis on the convergence mechanism between New Rural Cooperative Medical Scheme and Healthcare Financial Assistance Program. Chin. J. Health Policy.

[6] Babiarz K.S., Miller G., Yi H., Zhang L., Rozelle S. (2010). New evidence on the impact of China’s New Rural Cooperative Medical Scheme and its implications for rural primary healthcare: Multivariate difference-in-difference analysis. BMJ.

[7] Sun X., Jackson S., Carmichael G.A., Sleigh A.C. (2009). Prescribing behaviour of village doctors under China’s New Cooperative Medical Scheme. Soc. Sci. Med..

[8] Yip W., Hsiao W.C. (2009). Non-evidence-based policy: How effective is China’s new cooperative medical scheme in reducing medical impoverishment?. Soc. Sci. Med..

[9] Ma J., Xu J., Zhang Z., Wang J. (2016). New cooperative medical scheme decreased financial burden but expanded the gap of income-related inequity: Evidence from three provinces in rural China. Int. J. Equity Health.

[10] Ma X., Cen Y. (2017). Public Health Insurance System Reform and Its Impact on Health Service Utilization in Rural China: Evidence from CHNS 2000 and 2011. Chin. Stud..

[11] You X., Kobayashi Y. (2009). The new cooperative medical scheme in China. Health Policy.

[12] Lei X., Lin W. (2009). The new cooperative medical scheme in rural China: Does more coverage mean more service and better health?. Health Econ..

[13] Linder-Pelz S. (1982). Toward a theory of patient satisfaction. Soc. Sci. Med..

[14] Li J.G., Yang Z., Meng F. (2010). A Summary of patient satisfaction research and assessment tools. Mod. Hosp. Manag..

[15] Schoenfelder T., Klewer J., Kugler J. (2010). Factors associated with patient satisfaction in surgery: The role of patients’ perceptions of received care, visit characteristics, and demographic variables. J. Surg. Res..

[16] Wang H., Gu D., Dupre M.E. (2008). Factors associated with enrollment, satisfaction, and sustainability of the New Cooperative Medical Scheme program in six study areas in rural Beijing. Health Policy.

[17] Tasso K., Behar-Horenstein L.S., Aumiller A., Gamble K., Grimaudo N., Guin P., Ramey B. (2002). Assessing patient satisfaction and quality of care through observation and interview. Hosp. Top..

[18] McEachan R., Taylor N., Harrison R., Lawton R., Gardner P., Conner M. (2016). Meta-analysis of the reasoned action approach (RAA) to understanding health behaviors. Ann. Behav. Med..

[19] Kalolo A., Kibusi S.M. (2015). The influence of perceived behaviour control, attitude and empowerment on reported condom use and intention to use condoms among adolescents in rural Tanzania. Reprod. Health.

[20] Davis F.D. (1989). Perceived usefulness, perceived ease of use, and user acceptance of information technology. MIS Q..

[21] Fishbein M., Ajzen I. (1975). Belief, attitude, intention and behaviour: An introduction to theory and research. Philos. Rhetor..

[22] Davis F.D. (1993). User acceptance of information technology: System characteristics, user perceptions and behavioral imparts. Int. J. Man-Mach. Stud..

[23] Segars A.H., Grover V. (1993). Re-examining perceived ease of use and usefulness: A confirmatory factor analysis. MIS Q..

[24] Agarwal R., Karahanna E. (2000). Time flies when you’re having fun: Cognitive absorption and beliefs about information technology usage. MIS Q..

[25] Deng Z., Mo X., Liu S. (2014). Comparison of the middle-aged and older users’ adoption of mobile health services in China. Int. J. Med. Inform..

[26] Mackert M., Mabry-Flynn A., Champlin S., Donovan E.E., Pounders K. (2016). Health literacy and health information technology adoption: The potential for a new digital divide. J. Med. Internet Res..

[27] Buimer H.P., Tabak M., Van Velsen L., Van Der Geest T., Hermens H. (2017). Exploring Determinants of Patient Adherence to a Portal-Supported Oncology Rehabilitation Program: Interview and Data Log Analyses. JMIR Rehabilit. Assist. Technol..

[28] Davis F.D., Bagozzi R.P., Warshaw P.R. (1989). User acceptance of computer technology: A comparison of two theoretical models. Manag. Sci..

[29] Gulati R., Galino J. (2000). Get the right mix of bricks and clicks. Harv. Bus. Rev..

[30] Pan B., Yuan Z., Zou J., Cook D.M., Yang W. (2016). Elderly hospitalization and the New-type Rural Cooperative Medical Scheme (NCMS) in China: Multi-stage cross-sectional surveys of Jiangxi province. BMC Health Serv. Res..

[31] Yan Z., Wan D., Li L. (2011). Patient satisfaction in two Chinese provinces: Rural and urban differences. Int. J. Qual. Health Care.

[32] Bauer R.A. Consumer behavior as risk taking. Proceedings of the 43rd National Conference of the American Marketing Assocation.

[33] Malhotra N.K., Kim S.S., Agarwal J. (2004). Internet users’ information privacy concerns (IUIPC): The construct, the scale, and a causal model. Inf. Syst. Res..

[34] Van der Heijden H., Verhagen T., Creemers M. (2003). Understanding online purchase intentions: Contributions from technology and trust perspectives. Eur. J. Inf. Syst..

[35] Green D.T., Pearson J.M. (2011). Integrating website usability with the electronic commerce acceptance model. Behav. Inf. Technol..

[36] Gefen D., Karahanna E., Straub D.W. (2003). Trust and TAM in online shopping: An integrated model. MIS Q..

[37] Oster E., Thornton R. (2012). Determinants of technology adoption: Peer effects in menstrual cup take-up. J. Eur. Econ. Assoc..

[38] Venkatesh V., Morris M.G., Davis G.B., Davis F.D. (2003). User acceptance of information technology: Toward a unified view. MIS Q..

[39] Razee H., Whittaker M., Jayasuriya R., Yap L., Brentnall L. (2012). Listening to the rural health workers in Papua New Guinea–the social factors that influence their motivation to work. Soc. Sci. Med..

[40] Yoon C. (2009). The Effects of National Culture Values on Consumer Acceptance of E-Commerce: Online Shoppers in China. Inf. Manag..

[41] Limayem M., Hirt S.G. (2003). Force of Habit and Information Systems Usage: Theory and Initial Validation. J. Assoc. Inf. Syst..

[42] Gu D.X., Li P.P., Yang X.J. (2017). Investigating Users’ No-Show Behavior during using Hospital Online Appointment Registration System. J. Inf. Sci,.

[43] Song J., Zahedi F.M. (2006). Internet market strategies: Antecedents and implications. Inf. Manag..

[44] Dinev T., Hart P. (2006). An Extended Privacy Calculus Model for E-Commerce Transactions. Inf. Syst. Res..

[45] Bansal G., Gefen D. (2010). The impact of personal dispositions on information sensitivity, privacy concern and trust in disclosing health information online. Decis. Support Syst..

[46] Sun J., Sheng D., Gu D., Du J.T., Min C. (2017). Understanding link sharing tools continuance behavior in social media. Online Inf. Rev..

[47] Liang C., Gu D.X., Tao F.J., Jain H.K., Zhao Y., Ding B. (2017). Influence of mechanism of patient-accessible hospital information system implementation on doctor–patient relationships: A service fairness perspective. Inf. Manag..

[48] Hair J.F., Anderson R.E., Tatham R.L., Black W.C. (1998). Multivariate Data Analysis.

[49] Sanders N.R., Premus R. (2005). Modeling the relationship between firm IT capability, collaboration, and performance. J. Bus. Logist..

[50] Nunnally J.C., Bernstein I.H. (1978). Psychometric Theory.

[51] Bagozzi R.P. (1980). Causal Models in Marketing.

[52] Nunnally J.C., Bernstein I.H. (1994). Psychological Theory.

[53] Fornell C., Larcker D.F. (1981). Evaluating structural equation models with unobservable variables and measurement error. J. Mark. Res..

[54] Tung F.C., Chang S.C., Chou C.M. (2008). An extension of trust and TAM model with IDT in the adoption of the electronic logistics information system in HIS in the medical industry. Int. J. Med. Inform..

[55] Deng S.L., Guan X. (2016). Research on Influencing Factors of Users’ Health Information Acquisition Willingness Based on Question and Answer Platform. J. Inf. Sci..

